# Characterization of compliance phenotypes in COVID-19 acute respiratory distress syndrome

**DOI:** 10.1186/s12890-022-02087-8

**Published:** 2022-08-01

**Authors:** Lucia Zacchetti, Luca Longhi, Isabella Bianchi, Maria Di Matteo, Filippo Russo, Lucia Gandini, Leonardo Manesso, Martina Monti, Roberto Cosentini, Fabiano Di Marco, Stefano Fagiuoli, Lorenzo Grazioli, Paolo Gritti, Fabio Previdi, Michele Senni, Marco Ranieri, Luca Lorini, The Bergamo COVID-19 Group, Andrea Rota, Alessandra Martinelli, Paola Pugni, Antonella Marino, Giovanna Colombo, Marianna Damiani, Daniela Ferrari, Daniele Bonacina, Davide Corbella, Giancarla Poli, Diego Cantù, Francesco Ferri, Matteo Brivio, Ezio Bonanomi, Fabrizio Fabretti, Alberto Benigni, Pietro Brambillasca, Laura Scarpa, Federica Marchesi

**Affiliations:** 1 Department of Anesthesia, Emergency and Critical Care Medicine, Papa Giovanni XXIII Hospital, Piazza OMS 1, 24127 Bergamo, Italy; 2 Department of Anesthesia and Critical Care Medicine, University of Milan, Milan, Italy; 3 Emergency Medicine Department, Papa Giovanni XIII Hospital, Bergamo, Italy; 4 Department of Health Sciences, University of Milan, Milan, Italy; 5 Gastroenterology Hepatology and Transplantation Unit, Papa Giovanni XXIII Hospital, Bergamo, Italy; 6 Department of Medicine and Surgery, University of Milan Bicocca, Milan, Italy; 7 Department of Bioengineering, University of Bergamo, Bergamo, Italy; 8 Cardiovascular Department, Papa Giovanni XXIII Hospital, Bergamo, Italy; 9 Anesthesia and Intensive Care Medicine, Policlinico di Sant’Orsola, Alma Mater Studiorum University of Bologna, Bologna, Italy; 10 Respiratory Unit, Papa Giovanni XXIII Hospital, Bergamo, Italy

**Keywords:** Coronavirus, Acute respiratory distress syndrome, Prone positioning, Acute lung injury, Lung compliance, Sars coronavirus

## Abstract

**Background:**

Coronavirus disease 2019-associated acute respiratory distress syndrome (COVID-19 ARDS) seems to differ from the “classic ARDS”, showing initial significant hypoxemia in the face of relatively preserved compliance and evolving later in a scenario of poorly compliant lungs. We tested the hypothesis that in patients with COVID-19 ARDS, the initial value of static compliance of respiratory system (Crs) (1) depends on the previous duration of the disease (i.e., the fewer days of illness, the higher the Crs and vice versa) and (2) identifies different lung patterns of time evolution and response to prone positioning.

**Methods:**

This was a single-center prospective observational study. We enrolled consecutive mechanically ventilated patients with a diagnosis of COVID-19 who met ARDS criteria, admitted to intensive care unit (ICU). Patients were divided in four groups based on quartiles of initial Crs. Relationship between Crs and the previous duration of the disease was evaluated. Respiratory parameters collected once a day and during prone positioning were compared between groups.

**Results:**

We evaluated 110 mechanically ventilated patients with a diagnosis of COVID-19 who met ARDS criteria admitted to our ICUs. Patients were divided in groups based on quartiles of initial Crs. The median initial Crs was 41 (32–47) ml/cmH_2_O. No association was found between the previous duration of the disease and the initial Crs. The Crs did not change significantly over time within each quartile. Positive end-expiratory pressure (PEEP) and driving pressure were respectively lower and greater in patients with lower Crs. Prone positioning significantly improved PaO_2_/FiO_2_ in the 4 groups, however it increased the Crs significantly only in patients in lower quartile of Crs.

**Conclusions:**

In our cohort, the initial Crs is not dependent on the previous duration of COVID-19 disease. Prone positioning improves oxygenation irrespective to initial Crs, but it ameliorates respiratory mechanics only in patients with lower Crs.

**Supplementary Information:**

The online version contains supplementary material available at 10.1186/s12890-022-02087-8.

## Background

Severe COVID-19 pneumonia often determines an acute respiratory failure that fulfill criteria for Acute Respiratory Distress Syndrome (ARDS) [1]. Thus, since the outbreak of the pandemic, the proposed ventilatory strategies were consistent with those recommended by ARDS guidelines [2]. It has been suggested that COVID-19 ARDS may not adhere to a “classic” ARDS model but presents the unique pathophysiologic features of a significant hypoxemia in the face of relatively compliant lungs and that therefore a different approach to clinical management could be needed. Gattinoni et al. hypothesized that the early phase of COVID-19 ARDS is characterized by modest subpleural interstitial lung edema that leaves most of the lung aerated and preserved respiratory compliance. Subsequently, during the course of the disease, non-aerated portions of lung parenchyma may be prevalent leading to low compliance due to the increased lung inflammation and permeability [3]. Chest CT scan findings confirmed a time-related evolution of lung pattern infection [4]. The identification of different lung phenotypes of the same disease is clinically relevant because different patients may benefit from different therapeutic strategies.

This study tested the hypothesis that the initial value of static compliance of respiratory system (1) depends on the duration of the disease before ICU admission (i.e., the fewer days of illness, the higher the Crs and vice versa) and (2) identifies different lung patterns of time evolution (low versus high Crs) and response to prone positioning.

## Methods

This was a single-center retrospective observational study performed at the Papa Giovanni XXIII hospital, Bergamo (Italy). This study was conducted in accordance with the Declaration of Helsinki and approved by the ethics committee of Papa Giovanni XXIII hospital (approval number 72/20). Ethic committee of Papa Giovanni XIII Hospital of Bergamo waived the need of informed consent in the context of the COVID-19 outbreak.

All consecutive patients admitted to our ICUs in the period February 22nd and March 22nd 2020 with confirmed positive COVID-19 and acute respiratory failure (defined as PaO_2_/FiO_2_ < 300 and PEEP ≥ 5 cmH_2_O under invasive mechanical ventilation) were included.

Baseline clinical variables were collected at ICU admission. The following time intervals have been used to describe the duration of the disease before the ICU admission: (i) from the onset of COVID-19 symptoms to ICU admission, (ii) from hospital admission to ICU admission, (iii) from hospital admission to start of continuous positive airway pressure (CPAP) or non-invasive ventilation (NIV) and (iv) from start of CPAP or NIV to invasive mechanical ventilation (IMV).

All patients were sedated, paralyzed and ventilated in pressure or volume-control mode. Ventilatory settings were managed according to conventional protective settings [5]. Static compliance of respiratory system (Crs) was calculated as previously described [5]. Patients were divided based on quartiles of Crs: Q1 (Crs ≤ 25th percentile), Q2 (Crs > 25th and ≤ 50th percentile), Q3 (Crs > 50th and ≤ 75th percentile), Q4 (Crs > 75th percentile).

We collected the following respiratory parameters at baseline and daily for the first 14 days of ICU stay, or up to discharge or death: blood gas analysis, ventilation parameters [tidal volume/predicted body weight (V_T_/PBW), respiratory rate (RR), fraction of inspired oxygen (FiO_2_), positive end-expiratory pressure (PEEP)], lung mechanics [plateau pressure (Pplat), static compliance of respiratory system (Crs = Tidal Volume/Pplat-PEEP), driving pressure (dP = Pplat-PEEP)] and ventilator ratio (VR). VR, an index of impaired efficiency of ventilation, was calculated according the following formula: $VR = \frac{{\dot{V}_{Emeasured} \times PaCO_{2measured} }}{{\dot{V}_{Epredicted} \times PaCO_{2ideal} }}$[6].

Prone positioning was performed as previously described [7]. Briefly, patients were turned in prone position if presenting severe ARDS (defined as PaO_2_/FiO_2_ < 150 mmHg, with FiO_2_ ≥ 0.6, PEEP of ≥ 5 cmH_2_O and tidal volume of about 6 ml/Kg of predicted body weight); prone positioning was maintained for 16 consecutive hours, unless occurrence of major complications (i.e. endotracheal-tube obstruction, persistent oxygen desaturation, hemodynamic instability, cardiac arrest). To evaluate the effects of prone positioning, study variables were collected at three time points: before (supine pre), at the end (prone) and after 6 h (supine post) of each session of prone positioning. Ventilatory parameters, respiratory mechanics, gas exchanges and response to prone positioning and complications were compared among groups of Crs quartiles.

Occurrence of acute kidney injury (AKI), septic shock and barotrauma were recorded. A new pulmonary embolism was defined as positive chest CT angiogram with contrast confirmation of the embolism [8], or as a sudden new right ventricle overload in acute respiratory/hemodynamic deterioration not explained by other factors (i.e., pneumothorax, cardiac tamponade). Patient’s survival was evaluated 28-days and 6-months after ICU admission.

The data were extracted from the information systems using Label Studio and Apache Spark, open-source technologies capable to work with large amount of data with different representations. Data of uncertain values due to artifacts were reviewed. Patients’ personal information was analyzed in anonymous forms. All data generated or analyzed during the study are included in this published article in Additional files 1, 2, 3, 4, 5, 6 and 7.

Statistical analysis: descriptive statistics was used to summarize the data. Data with normal distribution were expressed as average ± standard deviation (SD), while ordinal data such as scores and data without normal distribution were expressed as median and interquartile range (IQR). Categorical data were expressed as count and percentage. The comparison between the 4 groups was performed using one-way Anova or Kruskal–Wallis test depending on parametric-nonparametric data and using the Fisher exact test for categorical variables. The comparison between groups over time was performed using the mixed-effects model followed by post-hoc correction. Linear regression was used for evaluating the association between compliance and time intervals. Prism and STATA were used to analyze the data. A *p* value < 0.05 was considered statistically significant.

## Results

### Characteristics and management of patients in ICU

From February 22nd to March 22nd 2020, 130 patients were admitted to our ICUs with a diagnosis of COVID-19 ARDS. One hundred and fifteen patients received invasive mechanical ventilation. Among these, only 110 patients had a value of static compliance of respiratory system reported within 48 h of ICU admission and were included in the analysis. A flow chart describing the patient’s inclusion process and grouping is shown Additional file 1: Fig. S1. Their clinical characteristics, laboratory findings and respiratory parameters are presented in Table 1*.* All patients presented bilateral infiltration at chest x-ray.

**Table 1:** Clinical characteristics, main laboratory findings and respiratory parameters of patients at ICU admission

Age (years)	60 ± 10
Male, n (%)	89 (81)
Body Mass Index	29 ± 5
Comorbidities, n (%)	
None	31 (27)
Hypertension	60 (54)
Coronary artery disease	12 (11)
Diabetes	20 (18)
Cancer	5 (4)
Chronic pulmonary disease	7 (6)
Chronic kidney disease	4 (4)
Symptoms, n (%)	
Fever	100 (91)
Dyspnea	86 (78)
Cough	62 (56)
Gastrointestinal	6 (5)
SOFA	6 ± 2
SAPS II	39 ± 11
Respiratory parameters	
Crs (ml/cmH_2_O)	41 (32–46)
PEEP (cmH_2_O)	15 (13–16)
PaO_2_/FiO_2_	110 (86–146)
PaCO_2_ (mmHg)	46 (37–54)
Vt/PBW (ml/Kg)	6.8 (6.3–7.4)
dP (cmH_2_O)	11 (10–13)
Laboratory tests	
WBC (10^3^/mcl)	10.9 ± 6.3
Platelets (10^3^/mcl)	212 ± 98
Bilirubin (mg/dl)	1.0 ± 0.8
Creatinine (mg/dl)	1.1 ± 0.9
Urea (mg/dl)	60 ± 34
Fibrinogen (mg/dl)	591 ± 191
CRP (mg/dl)	21 ± 10

Data are presented as mean ± SD. SOFA: Sequential Organ Failure Assessment Score. SAPS II: Simplified Acute Physiology Score. Crs: static compliance of respiratory system. PEEP: positive end-expiratory pressure. PaO_2_/FiO_2_: ratio of arterial oxygen partial pressure to fractional inspired oxygen. PaCO_2_: arterial carbon dioxide partial pressure. Vt/PBW: ratio of tidal volume to predicted body weight. dP: driving pressure. WBC: White Blood Cells. CRP: C Reactive Protein

### Groups of Crs quartiles: baseline characteristics and ventilator parameters over the first 14 days of ICU stay

**Fig. 1:**
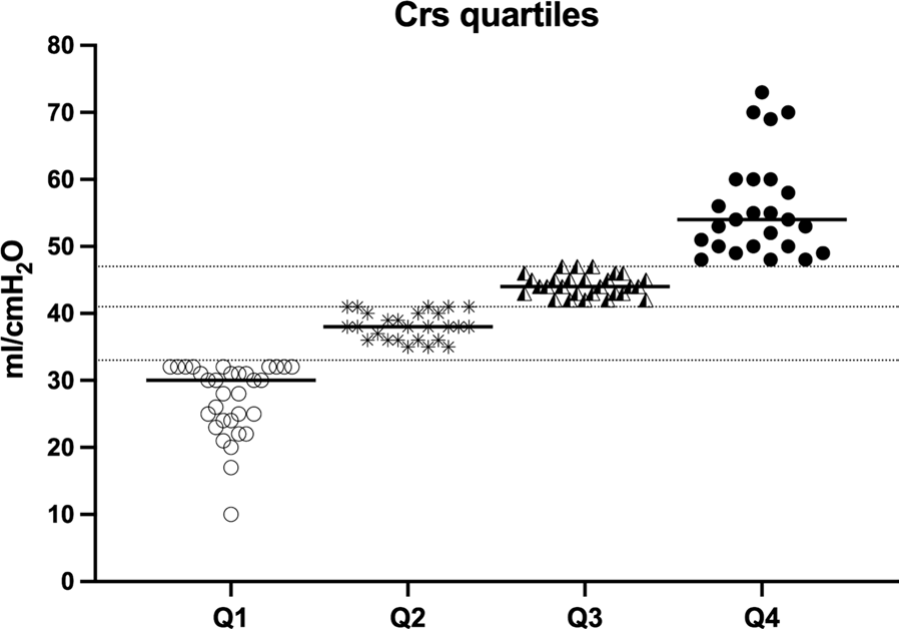
Static compliance of respiratory system (Crs) measured within 48 h after ICU admission. Legend: each symbol represents one patient. Based on the quartiles of compliance, patients were divided in four groups: Q1 (Crs ≤ 33 ml/cmH_2_O), Q2 (Crs > 33 and ≤ 41 ml/cmH_2_O), Q3 (Crs > 41 and ≤ 47 ml/cmH_2_0) and Q4 (Crs > 47 ml/cmH_2_O) groups. Crs: static compliance of respiratory system

The median initial Crs was 41 (32–47) ml/cmH_2_O ranging from 10 to 74 ml/cmH_2_O. The Crs was divided in quartiles: 32 patients (29%) had Crs ≤ 33 ml/cmH_2_0 (Q1-group); 26 patients (24%) had Crs > 33 and ≤ 41 ml/cmH_2_O (Q2-group); 27 patients (24%) had Crs > 41 and ≤ 47 ml/cmH_2_O (Q3-group); 25 patients (23%) had Crs > 47 ml/cmH_2_O (Q4-group) (Fig. 1). The prevalence of males progressively increased from Q1 to Q4 groups (*p* = 0.0008). No differences in terms of age, BMI, SOFA, SAPS II, comorbidities, and main respiratory therapies (immunomodulatory drugs, prone positioning and inhaled nitric oxide) were found between the four groups (Additional file 3: Table S1).

**Fig. 2:**
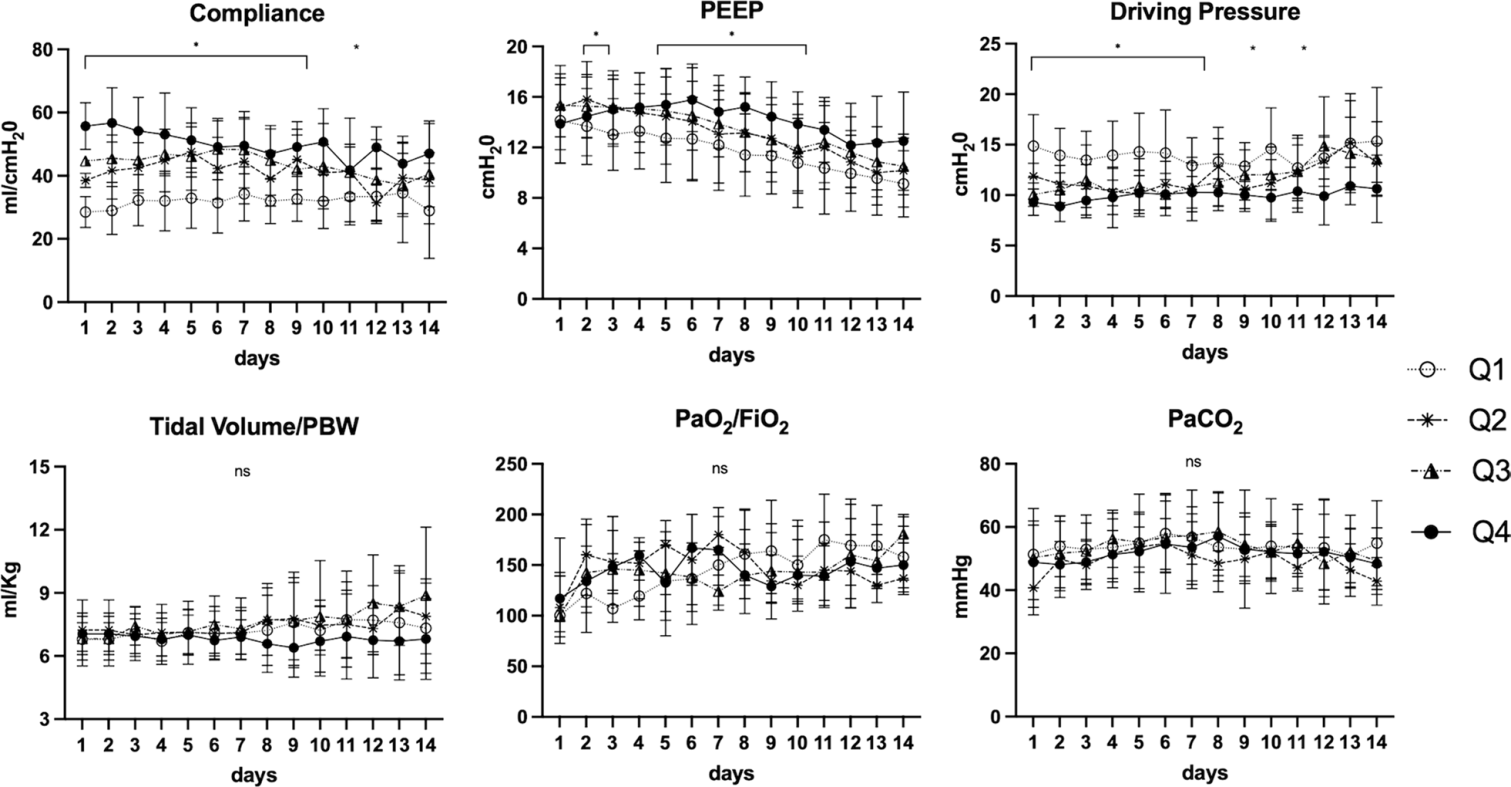
Respiratory parameters in the four groups of quartiles during the two weeks of ICU stay. Legend: data are presented as mean ± SD. *p < 0.05: post-hoc comparison between groups at a specified time point; ns: not statistically significant

Figure 2 shows the trend of respiratory variables during the first 14 days of ICU stay. The Crs did not change significantly over time within each quartile. Patients in the 4 Crs groups were ventilated at an average PEEP respectively of 12 ± 2 cm H_2_O in Q1-group, 13 ± 2 in Q2-group, 13 ± 2 in Q3-group and 14 ± 1 in Q4-group. PEEP was significantly different between groups Q1 and Q4 (*p* < 0.01). In the groups Q1, Q2 and Q3 the PEEP decreased significantly during the first 2 weeks. Driving pressure was respectively 14 ± 1 in Q1-group, 12 ± 1 in Q2-group, 12 ± 2 in Q3-group and 10 ± 1 in Q4-group. The driving pressure of Q1-group was significantly greater compared to that of other groups (*p* < 0.001). Driving pressure of Q4-group was also significantly lower than that of Q2 and Q3 groups (*p* < 0.01). No differences were seen in the other respiratory parameters (Additional file 4: Table S2).

### Relationship between the previous duration of the disease and initial Crs in the four Crs quartiles groups

No differences were found in the following time-intervals between the Crs quartiles groups: (1) from symptoms onset to ICU admission, (2) from hospital admission to ICU admission, (3) from hospital admission to CPAP/NIV and (4) from CPAP/NIV to mechanical ventilation (Table 2). Linear regression analysis showed that the duration of the disease had no effects on the initial Crs of the patients (Additional file 2: Fig. S2).

**Table 2:** Time intervals characterizing the duration of COVID disease in the groups of Crs quartiles

Time intervals, days	Q1	Q2	Q3	Q4	*p*
From symptoms onset to ICU admission	9 (7–16)	8 (7–11)	11 (6–14)	8 (6–12)	ns
From hospital admission to ICU admission	3 (1–6)	3 (1–4)	4 (2–6)	3 (2–5)	ns
From hospital admission to CPAP/NIV	1 (0–3)	0 (0–2)	1 (0–2)	1 (0–3)	ns
From CPAP/ NIV to IMV	3 (2–4)	2 (1–3)	3 (1–5)	2 (1–4)	ns

Data are presented as median (IQR). Crs: static compliance of respiratory system. CPAP: Continuous Positive Airway Pressure. NIV: non-invasive ventilation. IMV: invasive mechanical ventilation

### Response to prone positioning in patients within the four Crs quartiles groups

**Fig. 3:**
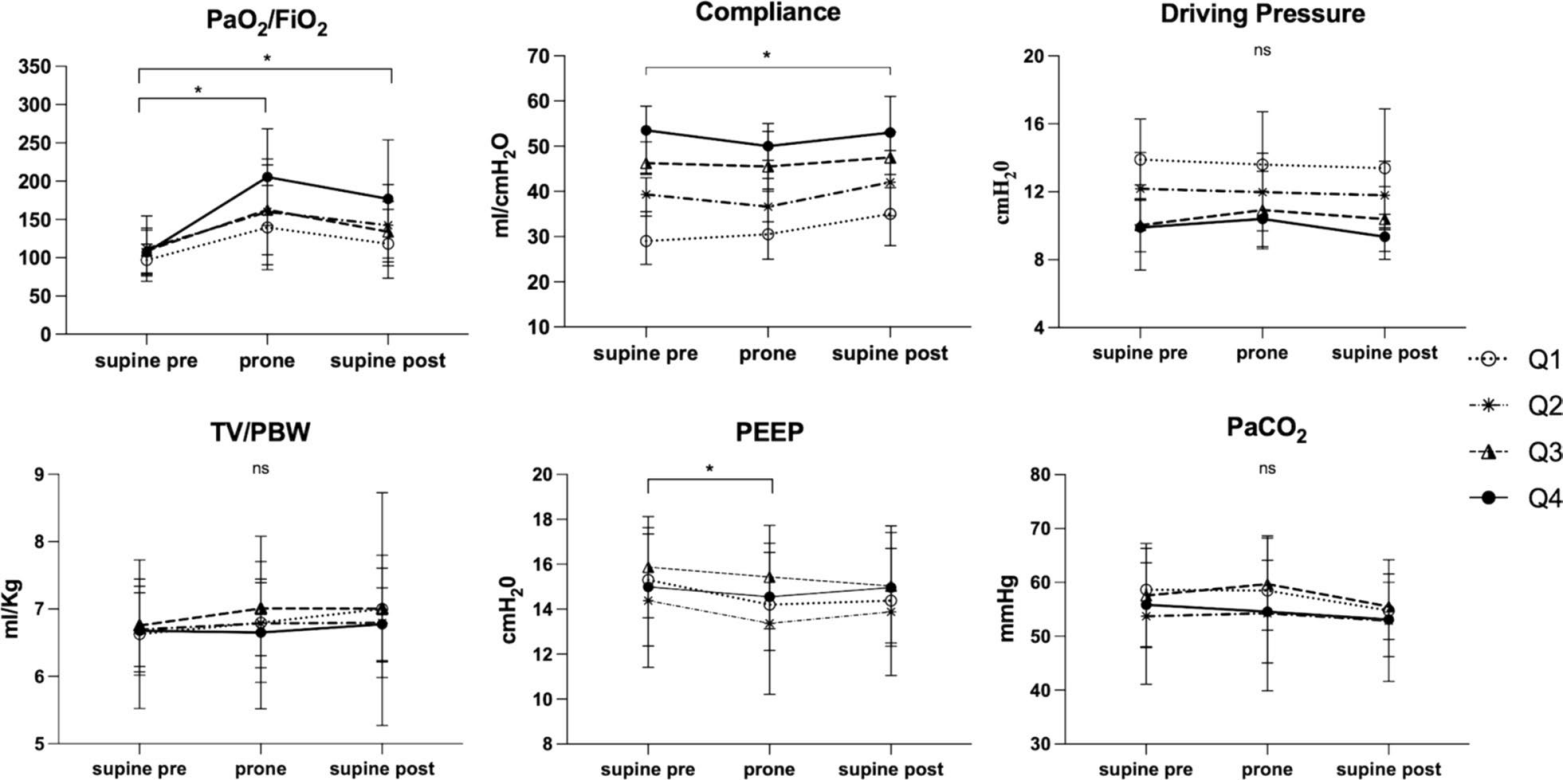
Changes of respiratory parameters during prone positioning. Legend: data are presented as mean ± SD. *p < 0.05: post-hoc comparison between groups at a specified time point. Supine pre: supine position before proning. Prone: 16 h after prone positioning. Supine post: 6-h after re-supination; ns: not statistically significant

Sixty-nine patients required prone positioning starting on day 2 (IQR 1–4) after ICU admission. Twenty-five patients (36%) were pronated once, while the remaining 44 needed more than a session. Figure 3 represents the changes of respiratory parameters during prone positioning. PaO_2_/FiO_2_ was significantly greater both in prone position and in supine position 6 h after pronation when compared to the baseline values in all groups (*p* < 0.05). The PaO_2_/FiO_2_ change associated with prone positioning was similar in the 4 groups (Additional file 5: Table S3). The PaO_2_/FiO_2_ improvement was consistently observed also when patients were subjected to repeated prone positioning sessions (Additional file 6: Table S4). Compliance increased significantly after prone positioning only in patients in the Q1 group. PEEP was reduced in prone position both in Q1 and in Q2 group (*p* < 0.05). No differences in other respiratory parameters were found (Additional file 5: Table S3).

### Complications in ICU during the first month in ICU, 28-days and 6-months outcome

AKI occurred in 68 patients (62%); 35% of them required CRRT. Twenty-nine (26%) had at least one episode of septic shock. Barotrauma was detected in 22 patients (20%). Pulmonary thromboembolism occurred in 16 patients (13%). Complications in ICU were not different within the 4 groups of quartiles (Additional file 7: Table S5).

Overall, 28-days and 6-month mortality occurred in 38 (34%) and 46 (42%) patients. Patients in Q1 group had a trend towards higher 28-days and 6-month mortality compared to other groups; however, this difference was not statistically significant (Additional file 7: Table S5).

## Discussion

Our study shows that, in patients affected by severe COVID-19 pneumonia and receiving mechanical ventilation: (1) the initial value of Crs ranged from low to normal values, demonstrating wide inter-individual heterogeneity; (2) the initial value of Crs was not associated with the duration of the disease before ICU admission; (3) all patients responded to prone positioning improving oxygenation but only patients with low Crs improved lung mechanics after prone positioning.

In our study we observed that the median Crs obtained within the first 48 h after starting invasive mechanical ventilation was 41 (IQR 32–47) being greater than usually observed in classic ARDS (median Crs approximately 30–32 ml/cmH_2_0 (IQR 23–43)) [9, 10] and consistent with those reported by Grasselli et al. [9] in a population of COVID-19-associated respiratory failure obtained within 24 h after ICU admission. Other authors reported case series of critically ill COVID-19 patients having a Crs 27—50 ml/cmH_2_O [11–13]. In our cohort, patients with highest and lowest Crs (Q1 and Q4) maintained a significant higher and lower compliance over the first 2 weeks in ICU. On the contrary, patients in the two intermediate quartiles of Crs overlapped their compliance during the whole period of observation. This result suggests that in our population, no transition from preserved to low compliance and vice versa occurred during the ICU stay.

According to the “classic” ARDS we would have expected worse oxygenation and PaCO_2_ in patients with low compliance. In contrast in our population, PaO_2_/FiO2 and PaCO_2_ were similar in the 4 groups over the first 2 weeks of ICU. These data suggest that the oxygenation is not only function of the amount of aerated lung, but other factors are involved. A major role could be played by the impairment of hypoxic vasoconstriction due to severe endothelial damage caused by the virus, well documented in the lungs of patients deceased for acute respiratory failure COVID-19-related [14] where the amount of alveolar capillary microthrombi is significantly more prevalent in patients with COVID-19 than in patients with H1N1-ARDS, thus suggesting that microthrombosis takes part in the pathogenesis of the disease.

Gattinoni et al. suggested that the Crs in COVID-19 patients may be function of the previous duration of the disease. In the early phase viral infection leads to modest subpleural interstitial lung edema that leaves most of the lung aerated and results in a preserved respiratory compliance (soft lung). Later in the course of the disease, the severity of infection itself and patient self-inflicted lung injury (P-SILI), lead to an increase in lung inflammation, lung permeability, interstitial and alveolar edema; non-aerated portions of lung parenchyma increase, causing an impairment of respiratory compliance (stiff lung) [3]. We could not confirm this hypothesis, since no relationship was observed between the time intervals considered to evaluate the previous duration of the disease and the value of initial compliance of respiratory system. Our data may suggest that Crs depends on factors other than the only time, such as the specific interaction between the host “vulnerability”, viral load and the degree of resulting inflammatory response [15–17]. Our data are consistent with those reported by Ferrando et al. that observed similar symptoms onset-mechanical ventilation and hospital admission-mechanical ventilation time-intervals in patients with reduced and normal Crs [18].

Overall, we observed an improvement in PaO_2_/FiO_2_ that lasted up to 6 h after prone positioning in all Crs quartiles groups with no differences in the degree of response between them.

Interestingly the compliance significantly increased only in the group with low Crs, indicating that these patients may have an amount of non-areated dependent recruitable lung that could be re-opened by prone positioning [3]. Similar to our results, Carsetti et al. [19] did not observed any change in Crs when prone positioning was used in patients with baseline Crs of 49 ± 9 ml/cmH_2_O, while Ziehr et al. [20] reported a Crs improvement when prone positioning was used in patients with baseline Crs of 35 ml/cmH_2_O (IQR 30–43). These results were comparable to prone positioning response of our patients with preserved and low Crs, respectively.

In our patients we observed a 28-days and 6-month mortality of 34% and 42%, respectively. In patients with classic ARDS low Crs is associated with increased mortality [7]. In our study patients in the four Crs quartiles showed similar ICU complications and mortality. Unfortunately, our study was under-powered to detect an association between mortality and compliance of respiratory system; however, we found a trend towards a higher mortality in the group of patients with lower compliance, suggesting that by increasing the size of our sample it would be possible to reach a statistically significant association.

Strengths of our study: evaluation of lung mechanics for 14 days, hence not limiting the observation of lung function to the hyper-acute phase only; characterization for the first time of Crs phenotypes in patients with COVID19; clarification that the phenotypes of reduced and normal Crs are not function of the previous duration of the disease and there is no transition from a normal to reduced Crs over the course of ICU stay; long-term outcome.

Limitations of our study: the study was limited to a single center and the sample size of each subgroup was relatively small. The choice of dividing patients in quartiles of baseline compliance was arbitrary and not ideal, however we were supported by other studies that have described in similar way the lung mechanics in ARDS population.

There were missing values in our dataset due to the fact that we were the first western hospital massively involved in COVID pandemic, with an overwhelming number of patients accessing to our emergency room-ICUs per day, making impossible to collect all the pre-defined data.

Furthermore, we could not perform lung CT scan in all patients, therefore we do not have enough exams to associate Crs and lung morphology. We did not evaluate the respiratory mechanics measuring the transpulmonary pressure.

## Conclusion

In our population the Crs was greater than that usually observed in classic ARDS. The Crs was not associated with the previous duration of the disease. In all patients prone positioning improved oxygenation, but it increased respiratory mechanics only in patients with baseline reduced respiratory Crs.

## Supplementary Information

**Additional file 1: Fig. S1.** Flow chat describing the patient’s inclusion process and grouping.

**Additional file 2: Fig. S2.** Association between time-intervals of the disease and the initial compliance of respiratory system.

**Additional file 3: Table S1.** Baseline characteristics, laboratory findings at ICU admission and additional therapies applied in the four groups of Crs quartiles.

**Additional file 4: Table S2.** Ventilator parameters, lung mechanics and gas exchanges in groups of compliance of respiratory system quartiles during the first 14 days of ICU stay.

**Additional file 5: Table S3.** Ventilatory setting, gas exchanges and lung mechanics during prone positioning in the four compliance of respiratory system quartiles.

**Additional file 6: Table S4.** Ventilatory setting, gas exchanges and lung mechanics during prone position, cycle-by-cycle, in the four groups of compliance of respiratory system quartiles.

**Additional file 7: Table S5.** Major complications occurred during the first 28-days of ICU stay.

## Data Availability

The datasets used and/or analysed during the current study are available from the corresponding author on reasonable request.

## Abbreviations

COVID-19 ARDS: Coronavirus disease 2019-associated acute respiratory distress syndrome
Crs: Static compliance of respiratory system
ICU: Intensive care unit
PEEP: Positive end-expiratory pressure
CT scan: Computed tomography scan
PaO_2_/FiO_2_: Ratio of arterial oxygen partial pressure to fractional inspired oxygen
CPAP: Continuous positive airway pressure
NIV: Non-invasive ventilation
IMV: Invasive mechanical ventilation
V_T_/PBW: Tidal volume/predicted body weight
RR: Respiratory rate
Pplat: Plateau pressure
dP: Driving pressure
VR: Ventilatory ratio
VEmeasured: Measured minute ventilation
VEpredicted: Predicted minute ventilation
PaCO2measured: Measured arterial carbon dioxide partial pressure
PaCO2predicted: Ideal arterial carbon dioxide partial pressure
AKI: Acute kidney injury
BMI: Body mass index
SOFA: Sequential organ failure assessment
SAPS II: Simplified acute physiology score
P-SILI: Patient self-inflicted lung injury
ANOVA: Analysis of variance
IQR: Interquartile range
SD: Standard deviation
