# Supplementary figures and images for: Characterization of compliance phenotypes in COVID-19 acute respiratory distress syndrome
Source: BMC Pulm Med. 2022 Aug 1;22:296. doi: 10.1186/s12890-022-02087-8 (PMC9341412; doi:10.1186/s12890-022-02087-8)

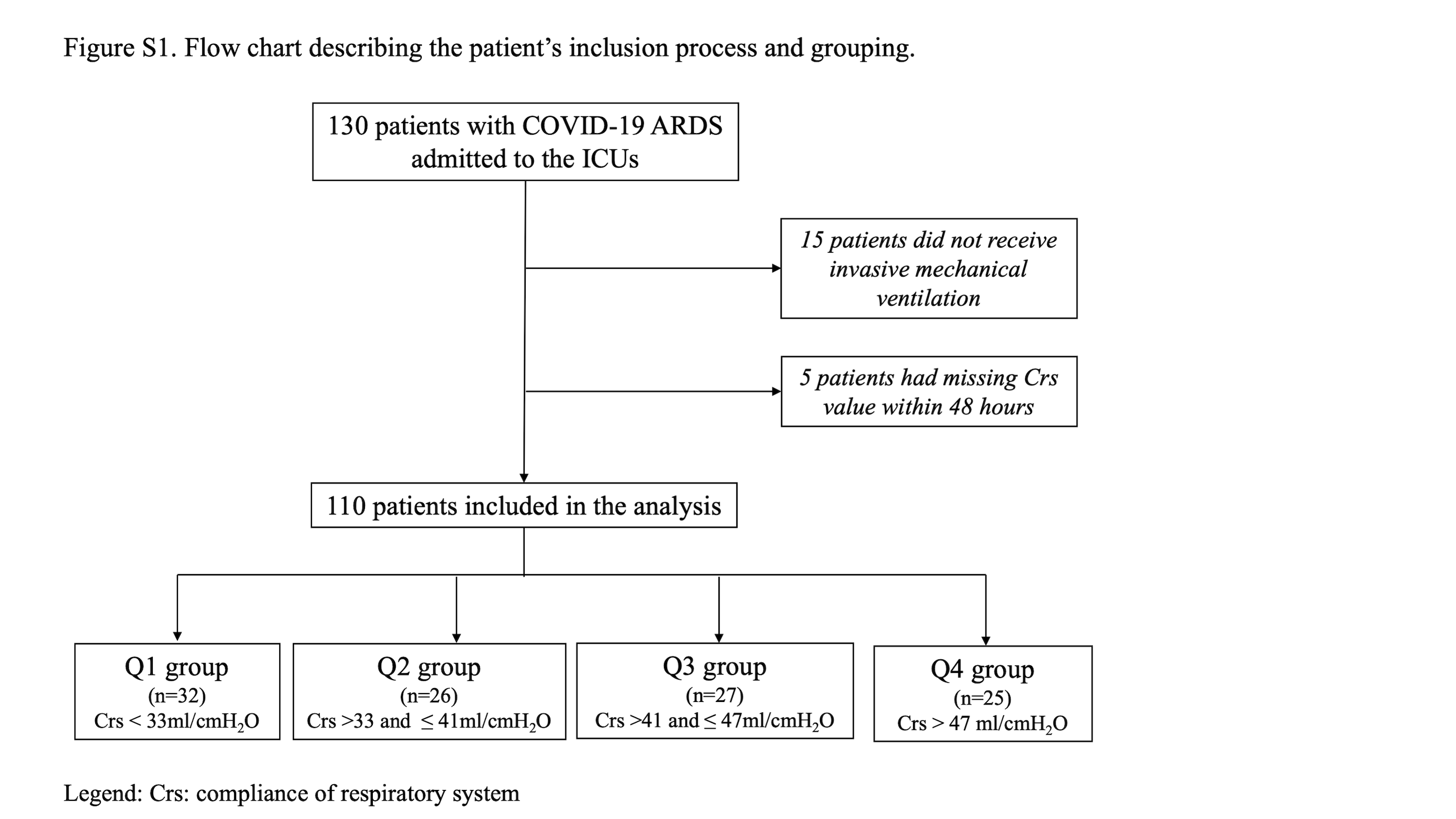

Supplement: Supplementary file 1 — Additional file 1: Fig. S1. Flow chat describing the patient’s inclusion process and grouping. [file 12890_2022_2087_MOESM1_ESM.tiff]

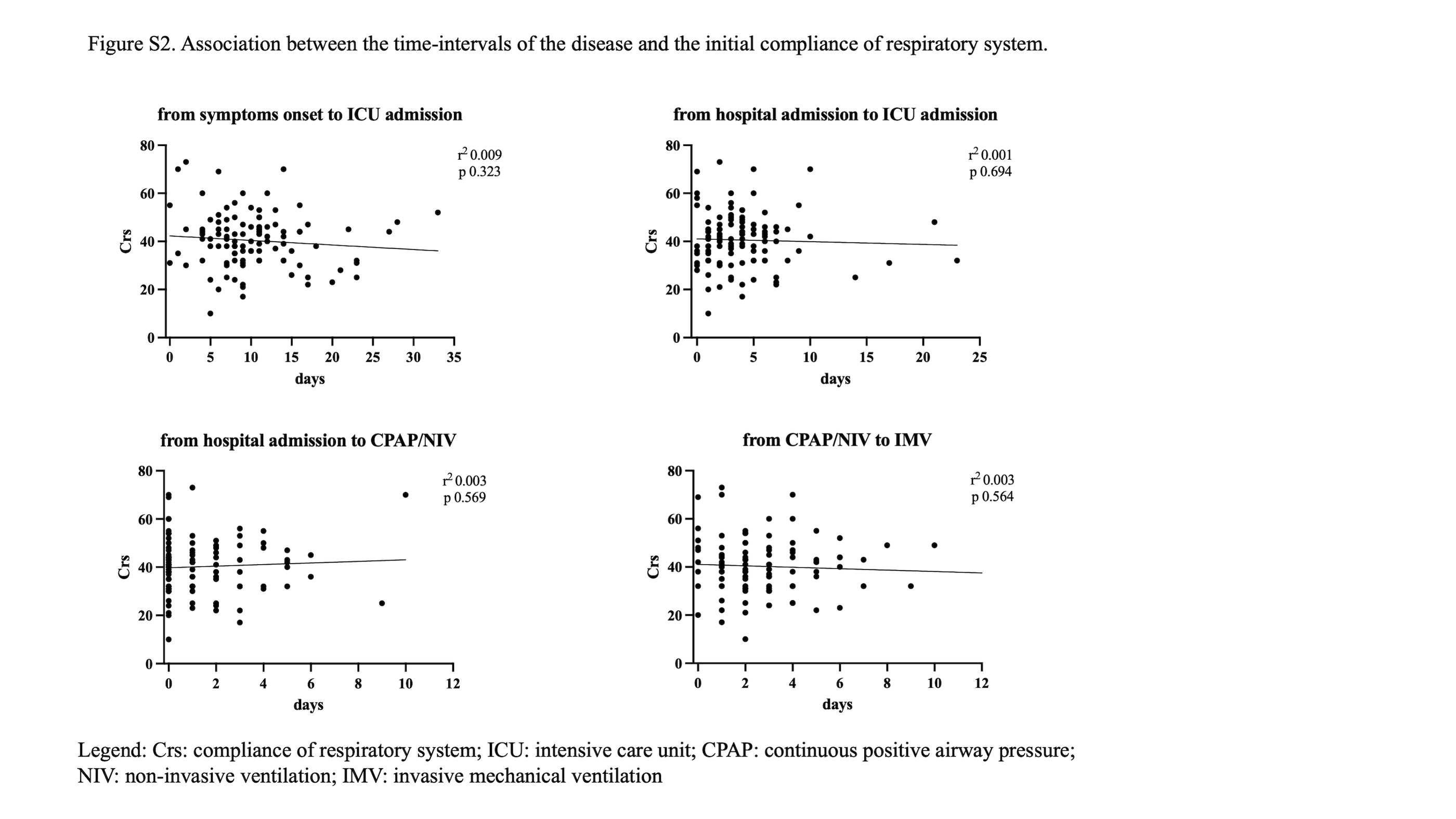

Supplement: Supplementary file 2 — Additional file 2: Fig. S2. Association between time-intervals of the disease and the initial compliance of respiratory system. [file 12890_2022_2087_MOESM2_ESM.tiff]
