# Supplementary material for: Characterization of compliance phenotypes in COVID-19 acute respiratory distress syndrome
Source: BMC Pulm Med. 2022 Aug 1;22:296. doi: 10.1186/s12890-022-02087-8 (PMC9341412; doi:10.1186/s12890-022-02087-8)
Supplement: Supplementary file 3 — Additional file 3: Table S1. Baseline characteristics, laboratory findings at ICU admission and additional therapies applied in the four groups of Crs quartiles. [file 12890_2022_2087_MOESM3_ESM.docx]

|  | Q1 (n=32) | Q2 (n=26) | Q3 (n=27) | Q4 (n=25) | p |
| --- | --- | --- | --- | --- | --- |
| Age (years) | 62 (52-69) | 62 (55-66) | 62 (54-69) | 59 (54-64) | ns |
| Male, n (%) | 20 (63) | 19 (73) | 26 (93) | 25 (100) | 0.0008 |
| Body Mass Index | 29 (25-33) | 28 (25-31) | 30 (26-33) | 28 (25-30) | ns |
| SOFA | 6 (4-8) | 5 (4-7) | 6 (4-8) | 5 (4-8) | ns |
| SAPS II | 39 (34-46) | 38 (33-46) | 37 (33-41) | 33 (30-43) | ns |
| Hypertension, n (%) | 16 (50) | 12 (46) | 17 (61) | 15 (60) | ns |
| Diabetes, n (%) | 2 (6) | 5 (19) | 8 (29) | 5 (20) | ns |
| COPD, n (%) | 2 (6) | 2 (8) | 2 (7) | 1 (4) | ns |
| CAD, n(%) | 2 (6) | 4 (15) | 3 (11) | 3 (12) | ns |
| Symptoms, n (%) |  |  |  |  |  |
| Fever | 29 (97) | 25 (96) | 25 (93) | 21 (84) | ns |
| Dyspnea | 25 (81) | 22 (85) | 22 (81) | 17 (68) | ns |
| Cough | 15 (48) | 14 (54) | 17 (65) | 16 (64) | ns |
| Gastrointestinal | 2 (6) | 2 (8) | 1 (4) | 1 (4) | ns |
| Laboratory tests |  |  |  |  |  |
| WBC (10^3^/mcl) | 12.1 (9.5-17.3) | 7.4 (5.1-12.7) | 8.7 (6.1-12.7) | 8.8 (6.9-10.7) | 0.02 |
| Lymphocytes (%) | 6.5 (2.5-9) | 6.5 (4-12) | 7 (6-9.5) | 7.5 (6.3-9.8) | ns |
| Platelets (10^3^/mcl) | 286 (128-238) | 198 (149-265) | 211 (159-297) | 176 (134-260) | ns |
| Bilirubin (mg/dl) | 0.8 (0.6-1.3) | 0.7 (0.4-0.9) | 0.9 (0.4-1.6) | 0.8 (0.7-1.4) | ns |
| Creatinine (mg/dl) | 0.9 (0.6-1.4) | 0.8 (0.7-1.1) | 0.8 (0.7-1.3) | 0.9 (0.7-1.2) | ns |
| Urea (mg/dl) | 52 (40-73) | 51 (37-61) | 56 (44-72) | 53 (31-69) | ns |
| Fibrinogen (mg/dl) | 698 (380-726) | 604 (520-776) | 614 (467-727) | 596 (445-743) | ns |
| INR | 1.2 (1.1-1.3) | 1.1 (1.1-1.2) | 1.1 (1.1-1.2) | 1.1 (1.1-1.2) | ns |
| CRP (mg/dl) | 23 (17-30) | 18 (13-24) | 24 (11-30) | 20 (11-29) | ns |
| Additional therapies, n (%) |  |  |  |  |  |
| Steroids | 18 (56) | 14 (54) | 15 (56) | 11 (44) | ns |
| Monoclonal antibodies | 10 (31) | 8 (31) | 10 (37) | 8 (32) | ns |
| Steroids plus monoclonal  antibodies | 5 (16) | 5 (19) | 8 (30) | 5 (20) | ns |
| Prone positioning | 20 (63) | 15 (58) | 20 (74) | 13 (54) | ns |
| Inhaled nitric oxide | 8 (25) | 6 (23) | 7 (26) | 10 (40) | ns |

Table S1. Baseline characteristics, laboratory findings at ICU admission and additional therapies applied in the four groups of Crs quartiles.

Legend: data are presented as mean±SD or as median (IQR). SOFA: Sequential Organ Failure Assessment Score. SAPS II: Simplified Acute Physiology Score. COPD: chronic obstructive pulmonary disease. CAD: coronary artery disease. WBC: white blood cell. INR: International Normalized Ratio. CRP: C Reactive Protein.
