# Supplementary material for: Characterization of compliance phenotypes in COVID-19 acute respiratory distress syndrome
Source: BMC Pulm Med. 2022 Aug 1;22:296. doi: 10.1186/s12890-022-02087-8 (PMC9341412; doi:10.1186/s12890-022-02087-8)
Supplement: Supplementary file 4 — Additional file 4: Table S2. Ventilator parameters, lung mechanics and gas exchanges in groups of compliance of respiratory system quartiles during the first 14 days of ICU stay. [file 12890_2022_2087_MOESM4_ESM.docx]

Table S2. Ventilator parameters, lung mechanics and gas exchanges in groups of compliance of respiratory system quartiles during the first 14 days of ICU stay.

|  | Q1 | | | | Q2 | | | | Q3 | | | | Q4 | | | |
| --- | --- | --- | --- | --- | --- | --- | --- | --- | --- | --- | --- | --- | --- | --- | --- | --- |
| day of ICU stay | 1 | 3 | 7 | 14 | 1 | 3 | 7 | 14 | 1 | 3 | 7 | 14 | 1 | 3 | 7 | 14 |
| *count of patients* | 32 | 29 | 23 | 13 | 26 | 23 | 15 | 10 | 27 | 27 | 24 | 11 | 25 | 23 | 19 | 13 |
| Crs (ml/cmH_2_O) | 28±5^°^#^ | 32±8^°^#^ | 34±9^^#^ | 29±15 | 38±2^*^#^ | 42±8^*#^ | 44±13 | 39±9 | 45±1^*°#^ | 45±9^*#^ | 48±12^*^ | 41±16 | 56±7^*°^^ | 54±11^*°^^ | 49±9^*^ | 47±10 |
| PEEP (cmH_2_O) | 14±3 | 13±3^^^ | 12±4^#^ | 9±3 | 15±3 | 15±3 | 13±4 | 10±3 | 15±2 | 15±2^*^ | 14±3 | 10±2 | 14±3 | 15±3 | 15±3^*^ | 12±4 |
| Vt/PBW (ml/Kg) | 6.8±1.3 | 7.1±1.3 | 7.1±1.2 | 7.3±2.1 | 7.2±1.4 | 7.0±1.0 | 7.1±1.0 | 7.9±1.8 | 6.8±0.8 | 7.4±0.9 | 7.3±1.5 | 8.9±3.2 | 7.0±0.8 | 6.9±0.8 | 6.9±0.8 | 6.8±1.9 |
| RR (breath/min) | 23±5 | 23±6 | 22±6 | 20±6 | 22±4 | 23±4 | 23±4 | 20±3 | 23±4 | 24±4 | 24±4 | 19±5 | 22±6 | 22±4 | 23±7 | 19±8 |
| PaO_2_/FiO_2_ | 117±50 | 133±62 | 151±65 | 169±62 | 115±46 | 161±47 | 169±51 | 144±31 | 121±62 | 153±41 | 133±46^#^ | 171±50 | 143±71 | 159±63 | 172±53^^^ | 159±53 |
| PaCO_2_ | 51±11^°^ | 53±11 | 57±15 | 55±13^°^ | 41±8^*^ | 48±8 | 51±11 | 43±7^*^ | 50±16 | 52±9 | 57±9 | 49±11 | 49±12 | 49±7 | 53±11 | 48±8 |
| dP (cmH_2_O) | 15±3^°^#^ | 13±3^°#^ | 13±3 | 15±5^#^ | 12±1^*^#^ | 11±2^*^ | 11±3 | 13±2 | 10±1^*°^ | 11±3 | 11±2 | 14±4 | 9±1^°*^ | 9±2^*^ | 10±2^*^ | 11±3 |
| VR | 2.1±0.7 | 2.3±0.7 | 2.5±1.2 | 2.2±0.8 | 1.7±0.3^^^ | 2.0±1.4^^^ | 2.1±0.5 | 1.9±0.4 | 2.3±0.5^°^ | 2.5±0.6^°^ | 2.5±0.6 | 2.3±0.7 | 2.0±0.6 | 2.1±0.6 | 2.3±1.1 | 1.7±1.0 |

Legend: data are presented as mean±SD. PEEP: Positive End-Expiratory Pressure. Vt/PBW: tidal volume/Predicted Body Weight. RR: respiratory rate. PaO_2_: arterial partial pressure of oxygen; FiO_2_: fraction of inspired oxygen. PaCO_2_: arterial partial pressure of Carbon Dioxide. dP: driving pressure. Pplat: plateau pressure. VR: ventilatory ratio. At a specified time point: * 0<0.05: post-hoc analysis versus Q1; ° post-hoc analysis versus Q2; ^ post-hoc analysis versus Q3; ^#^ post-hoc analysis versus Q4.
