# Supplementary material for: Characterization of compliance phenotypes in COVID-19 acute respiratory distress syndrome
Source: BMC Pulm Med. 2022 Aug 1;22:296. doi: 10.1186/s12890-022-02087-8 (PMC9341412; doi:10.1186/s12890-022-02087-8)
Supplement: Supplementary file 5 — Additional file 5: Table S3. Ventilatory setting, gas exchanges and lung mechanics during prone positioning in the four compliance of respiratory system quartiles. [file 12890_2022_2087_MOESM5_ESM.docx]

|  | Q1 (n=20) | | | Q2 (n=15) | | | Q3 (n=20) | | | Q4 (n=14) | | |
| --- | --- | --- | --- | --- | --- | --- | --- | --- | --- | --- | --- | --- |
|  | supine pre | prone | supine post | supine pre | prone | supine post | supine pre | prone | supine post | supine pre | prone | supine post |
| PaO_2_/FiO_2_ | 97±21 | 139±55^*†^ | 118±45^°^ | 112±43 | 160±69^*^ | 142±53^°^ | 109±29 | 162±59^*^ | 134±39^°^ | 107±29 | 205±63^*†^ | 177±77^°^ |
| PCO_2_ (cmH_2_O) | 59±12 | 58±12 | 55±12 | 54±13 | 54±14 | 53±11 | 57±10 | 60±8 | 55±6 | 56±8 | 55±9 | 53±7 |
| Vt/PBW (ml/Kg) | 6.6±1.1 | 6.8±1.3 | 7.0±1.7 | 6.7±0.5 | 6.8±0.7 | 6.8±0.8 | 6.8±0.7 | 7.0±0.7 | 7.0±0.8 | 6.7±0.7 | 6.6±0.7 | 6.8±0.5 |
| PEEP (cmH_2_O) | 15±3 | 14±3^*^ | 14±3 | 14±3 | 13±3^*^ | 14±3 | 16±2 | 15±2 | 15±3 | 15±3 | 14±2 | 15±2 |
| Crs (ml/cmH_2_O) | 30±8 | 32±8 | 35±11^°^ | 39±6 | 40±8 | 40±8 | 49±7 | 46±8 | 49±9 | 52±10 | 49±8 | 55±8 |
| dP (cmH_2_O) | 14±2 | 14±3 | 13±4 | 12±2 | 12±2 | 12±2 | 10±2 | 11±2 | 10±2 | 10±2 | 10±2 | 9±1 |
| VR | 2.4±0.7 | 2.5±0.6 | 2.5±0.5 | 2.0±0.3 | 2.1±0.4 | 2.1±0.5 | 2.7±0.6 | 2.9±0.6 | 2.7±0.5 | 2.5±0.5 | 2.3±0.5 | 2.3±0.5 |

Table S3. Ventilatory setting, gas exchanges and lung mechanics during prone positioning in the four compliance of respiratory system quartiles.

Legend: supine pre = before prone positioning; prone = at the end of prone positioning; supine post = at 6 hours after prone positioning. Data are presented as mean±SD.*p<0.05: post-hoc comparison prone versus supine_pre; °p<0.05: post-hoc comparison supine post versus supine pre; ^†^p<0.05: post-hoc comparison between different groups in the same position. PaO_2_: arterial partial pressure of oxygen; FiO_2_: fraction of inspired oxygen. PaCO_2_: arterial partial pressure of carbon dioxide. Vt/PBW: tidal volume/Predicted Body Weight. PEEP: Positive End-Expiratory Pressure. Crs: static compliance of respiratory system. dP: driving pressure. VR: ventilatory ratio.
