# Supplementary material for: Characterization of compliance phenotypes in COVID-19 acute respiratory distress syndrome
Source: BMC Pulm Med. 2022 Aug 1;22:296. doi: 10.1186/s12890-022-02087-8 (PMC9341412; doi:10.1186/s12890-022-02087-8)
Supplement: Supplementary file 6 — Additional file 6: Table S4. Ventilatory setting, gas exchanges and lung mechanics during prone position, cycle-by-cycle, in the four groups of compliance of respiratory system quartiles. [file 12890_2022_2087_MOESM6_ESM.docx]

Table S4. Ventilatory setting, gas exchanges and lung mechanics during prone position, cycle-by-cycle, in the four groups of compliance of respiratory system quartiles.

|  | 1° cycle (n=69) | | | | | | | | | | | | 2° cycle (n=44) | | | | | | | | | | | |
| --- | --- | --- | --- | --- | --- | --- | --- | --- | --- | --- | --- | --- | --- | --- | --- | --- | --- | --- | --- | --- | --- | --- | --- | --- |
|  | Q1 (n=20) | | | Q2 (n=15) | | | Q3 (n=20) | | | Q4 (n=14) | | | Q1 (n=15) | | | Q2 (n=9) | | | Q3 (n=11) | | | Q4 (n=9) | | |
|  | supine pre | prone | supine post | supine pre | prone | supine post | supine pre | prone | supine post | supine pre | prone | supine post | supine pre | prone | supine post | supine pre | prone | supine post | supine pre | prone | supine post | supine pre | prone | supine post |
| PaO_2_/FiO_2_ | 89±28 | 125±49^*^ | 113±36 | 104±54 | 159±86 | 140±57 | 105±41 | 173±82^*^ | 129±42^°^ | 100±34 | 222±84^*^ | 153±82^°^ | 109±30 | 161±96 | 140±61 | 125±43 | 157±49^*^ | 150±70 | 118±32 | 169±80 | 124±34 | 125±31 | 185±52^*^ | 133±30 |
| PaCO_2_ (cmH_2_O) | 58±11 | 58±8 | 56±11 | 52±7 | 53±16 | 53±14 | 56±13 | 60±12 | 53±6 | 53±9 | 54±11 | 53±8 | 55±12 | 56±9 | 63±11 | 54±17 | 50±7 | 46±6 | 57±6 | 57±8 | 60±5 | 59±16 | 54±6 | 55±9 |
| Vt/PBW (ml/Kg) | 6.5±1.0 | 6.8±0.7 | 6.7±1.4 | 6.7±0.8 | 6.7±0.9 | 7.0±0.8 | 6.7±1.0 | 6.7±0.6 | 6.9±0.9 | 6.8±0.6 | 7.1±0.8 | 7.1±0.7 | 6.7±1.4 | 6.9±1.6 | 6.9±1.0 | 6.9±0.9 | 6.8±1.0 | 7.3±1.4 | 7.1±1.3 | 6.5±1.0 | 7.1±1.4 | 6.9±0.5 | 6.9±0.5 | 7.0±0.6 |
| PEEP (cmH_2_O) | 15±4 | 14±3 | 15±3 | 15±3 | 14±3 | 15±3 | 16±2 | 15±2 | 15±3 | 16±3 | 15±2 | 15±3 | 15±3 | 16±3 | 15±3 | 15±3 | 14±3 | 14±3 | 17±1 | 17±2 | 16±3 | 15±4 | 15±3 | 15±3 |
| RR  (breath/min) | 24±5 | 26±4^*^ | 25±4 | 21±3 | 22±3 | 22±4 | 24±4 | 26±3 | 25±5 | 23±3 | 23±4 | 24±3 | 24±4 | 25±4 | 25±5 | 22±4 | 23±3 | 22±5 | 26±3 | 26±3 | 27±2 | 24±3 | 24±3 | 24±4 |
| Crs (ml/cmH_2_O) | 30±7 | 33±9^*^ | 36±11^°^ | 38±5 | 40±8 | 40±9 | 47±5 | 46±9 | 50±16 | 51±10 | 54±13 | 57±9 | 31±10 | 34±8 | 37±12^°^ | 41±11 | 42±7 | 42±5 | 54±18 | 50±6 | 49±5 | 55±12 | 49±6 | 57±11 |
| dP (cmH_2_O) | 14±3 | 14±3 | 13±3 | 12±2 | 12±2 | 12±2 | 10±2 | 11±3 | 10±2 | 10±4 | 10±2 | 9±1 | 13±3 | 12±3 | 12±3 | 12±3 | 11±3 | 11±2 | 9±2 | 9±1 | 9±1 | 9±2 | 10±1 | 9±2 |
| Pplat (cmH_2_O) | 29±3 | 28±3 | 29±4 | 28±3 | 27±3 | 26±3 | 27±3 | 27±3 | 26±2 | 26±3 | 26±3 | 25±2 | 27±3 | 28±4 | 27±3 | 28±3 | 25±3 | 25±3 | 27±2 | 27±1 | 27±2 | 26±2 | 26±3 | 25±3 |
| VR | 2.3±0.7 | 2.7±0.5 | 2.3±0.5 | 1.9±0.3 | 2.0±0.4 | 2.0±0.5 | 2.5±0.6 | 2.9±0.7 | 2.6±0.5 | 2.3±0.6 | 2.3±0.4 | 2.3±0.4 | 2.2±0.9 | 2.4±0.6 | 2.7±0.9 | 2.3±0.8 | 2.1±0.5 | 2.3±0.7 | 2.7±0.5 | 2.9±0.6 | 2.5±0.9 | 2.7±1.1 | 2.4±0.5 | 2.5±0.6 |

|  | 3° cycle (n=21) | | | | | | | | | | | |
| --- | --- | --- | --- | --- | --- | --- | --- | --- | --- | --- | --- | --- |
|  | Q1 (n=5) | | | Q2 (n=4) | | | Q3 (n=8) | | | Q4 (n=4) | | |
|  | supine pre | prone | supine post | supine pre | prone | supine post | supine pre | prone | supine post | supine pre | prone | supine post |
| PaO_2_/FiO_2_ | 121±43 | 158±33 | 166±45 | 147±76 | 174±23 | 165±35 | 119±33 | 190±75 | 157±51 | 106±48 | 164±81 | 149±89 |
| PaCO_2_ (cmH_2_O) | 73±16 | 76±19 | 71±9 | 43±4 | 45±3 | 57±0 | 59±15 | 65±14 | 63±9 | 57±6 | 51±5 | 53±7 |
| Vt/PBW (ml/Kg) | 6.9±1.2 | 7.1±1.2 | 7.3±0.3 | 7.4±1.6 | 6.2±0.5 | 6.2±0.2 | 7.0±1.2 | 6.7±0.7 | 7.3±1.1 | 6.7±0.5 | 7.1±0.5 | 7.0±0.9 |
| PEEP (cmH_2_O) | 16±1 | 15±2 | 14±1 | 15±3 | 16±2 | 16±4 | 17±2 | 17±1 | 17±2 | 15±2 | 15±2 | 14±2 |
| RR  (breath/min) | 24±4 | 27±1 | 22±4 | 21±6 | 23±4 | 21±1 | 26±4 | 26±4 | 26±4 | 25±2 | 23±5 | 21±5 |
| Crs (ml/cmH_2_O) | 30±13 | 30±9 | 31±11 | 43±6 | 47±15 | 44±8 | 52±13 | 50±12 | 48±8 | 58±13 | 57±20 | 56±12 |
| dP (cmH_2_O) | 13±4 | 13±3 | 14±4 | 11±2 | 10±3 | 10±2 | 10±2 | 10±2 | 10±1 | 8±2 | 9±3 | 10±2 |
| Pplat (cmH_2_O) | 29±3 | 28±2 | 28±5 | 26±3 | 26±3 | 26±2 | 27±2 | 27±3 | 27±3 | 24±3 | 24±4 | 26±1 |
| VR | 2.3±0.6 | 3.0±1 | 2.8±0.7 | 2.0±0 | 2.5±1.0 | 3.0±1 | 2.8±1.0 | 2.8±0.8 | 2.6±0.7 | 2.8±0.8 | 2.2±0.8 | 3.0±0.8 |

Legend: supine pre = before prone positioning; prone = at the end of prone positioning; supine post = at 6 hours after prone positioning. Data are presented as mean ± SD. *p<0.05: post-hoc comparison prone versus supine pre. °p<0.05: post-hoc comparison supine post versus supine pre. PaO_2_: arterial partial pressure of oxygen; FiO_2_: fraction of inspired oxygen. PaCO_2_: arterial partial pressure of carbon dioxide. Vt/PBW: tidal volume/Predicted Body Weight. Peep: Positive End-Expiratory Pressure. Crs: static compliance of respiratory system. dP: driving pressure. Pplat: plateau pressure. VR: ventilatory ratio.
