# Supplementary material for: Characterization of compliance phenotypes in COVID-19 acute respiratory distress syndrome
Source: BMC Pulm Med. 2022 Aug 1;22:296. doi: 10.1186/s12890-022-02087-8 (PMC9341412; doi:10.1186/s12890-022-02087-8)
Supplement: Supplementary file 7 — Additional file 7: Table S5. Major complications occurred during the first 28-days of ICU stay. [file 12890_2022_2087_MOESM7_ESM.docx]

|  | Q1 (n=32) | Q2 (n=26) | Q3 (n=27) | Q4 (n=25) | p |
| --- | --- | --- | --- | --- | --- |
| Major complications, n (%) |  |  |  |  |  |
| Acute Kidney Injury | 18 (56) | 14 (56) | 19 (70) | 17 (68) | ns |
| CRRT | 5 (15) | 5 (19) | 6 (22) | 8 (32) | ns |
| Septic shock | 8 (25) | 7 (27) | 8 (30) | 6 (24) | ns |
| Barotrauma | 10 (31) | 2 (8) | 4 (15) | 6 (24) | ns |
| Pulmonary  thromboembolism | 7 (2) | 2 (8) | 4 (15) | 3 (12) | ns |
| 28-days mortality | 14 (44) | 6 (23) | 10 (37) | 8 (32) | ns |
| 6-month mortality | 17 (55) | 9 (35) | 11 (41) | 8 (32) | ns |

Table S5. Major complications occurred during the first 28-days of ICU stay.

Legend: data are presented as count and (%). CRRT: Continuous renal replacement therapies. LOS: length of stay; ICU: intensive care unit.
